# ^31^P-NMR Metabolomics Revealed Species-Specific Use of Phosphorous in Trees of a French Guiana Rainforest

**DOI:** 10.3390/molecules25173960

**Published:** 2020-08-31

**Authors:** Albert Gargallo-Garriga, Jordi Sardans, Joan Llusià, Guille Peguero, Dolores Asensio, Romà Ogaya, Ifigenia Urbina, Leandro Van Langenhove, Lore T. Verryckt, Elodie A. Courtois, Clément Stahl, Oriol Grau, Otmar Urban, Ivan A. Janssens, Pau Nolis, Miriam Pérez-Trujillo, Teodor Parella, Josep Peñuelas

**Affiliations:** 1 CSIC, Global Ecology Unit CREAF-CSIC-UAB, Bellaterra, 08193 Catalonia, Spain; j.sardans@creaf.uab.cat (J.S.); guille.peguero@gmail.com (G.P.); grau.oriol@gmail.com (O.G.); Josep.Penuelas@uab.cat (J.P.); 2 CREAF, Cerdanyola del Vallès, 08193 Catalonia, Spain; j.llusia@creaf.uab.cat (J.L.); loles@creaf.uab.cat (D.A.); r.ogaya@creaf.uab.cat (R.O.); ifigeniaurbinab@gmail.com (I.U.); 3 Global Change Research Institute, Czech Academy of Sciences, Belidla 986/4a, CZ-60300 Brno, Czech Republic; urban.o@czechglobe.cz; 4 Department of Biology, University of Antwerp, BE-2610 Wilrijk, Belgium; leandro.vanlangenhove@uantwerpen.be (L.V.L.); Lore.Verryckt@uantwerpen.be (L.T.V.); courtoiselodie@gmail.com (E.A.C.); ivan.janssens@uantwerpen.be (I.A.J.); 5 Laboratoire Ecologie, évolution, Interactions des Systèmes Amazoniens (LEEISA), Université de Guyane, CNRS, IFREMER, 97300 Cayenne, French Guiana; 6 INRA, UMR EcoFoG, CNRS, Cirad, AgroParisTech, Université des Antilles, Université de Guyane, 97310 Kourou, France; Clement.Stahl@ecofog.gf; 7 Cirad, UMR EcoFoG (AgroParisTech, CNRS, Inra, Univ Antilles, Univ Guyane), Campus Agronomique, 97310 Kourou, French Guiana; 8 Servei de Ressonància Magnètica Nuclear, Universitat Autònoma de Barcelona, E-08193 Bellaterra, Catalonia, Spain; Pau.Nolis@uab.cat (P.N.); miriam.perez@uab.cat (M.P.-T.); Teodor.Parella@uab.cat (T.P.)

**Keywords:** ^31^P-NMR metabolic profiling, Iceland, tropical lowland, P-containing compounds, species-specific P-use niches

## Abstract

Productivity of tropical lowland moist forests is often limited by availability and functional allocation of phosphorus (P) that drives competition among tree species and becomes a key factor in determining forestall community diversity. We used non-target ^31^P-NMR metabolic profiling to study the foliar P-metabolism of trees of a French Guiana rainforest. The objective was to test the hypotheses that P-use is species-specific, and that species diversity relates to species P-use and concentrations of P-containing compounds, including inorganic phosphates, orthophosphate monoesters and diesters, phosphonates and organic polyphosphates. We found that tree species explained the 59% of variance in ^31^P-NMR metabolite profiling of leaves. A principal component analysis showed that tree species were separated along PC 1 and PC 2 of detected P-containing compounds, which represented a continuum going from high concentrations of metabolites related to non-active P and P-storage, low total P concentrations and high N:P ratios, to high concentrations of P-containing metabolites related to energy and anabolic metabolism, high total P concentrations and low N:P ratios. These results highlight the species-specific use of P and the existence of species-specific P-use niches that are driven by the distinct species-specific position in a continuum in the P-allocation from P-storage compounds to P-containing molecules related to energy and anabolic metabolism.

## 1. Introduction

Nitrogen (N) and phosphorus (P) are essential nutrients for photosynthetic carbon assimilation and represent the most common net primary productivity limiting nutrients in terrestrial ecosystems. Nitrogen availability constrains plant productivity in many temperate and boreal forests by limiting leaf initiation and expansion [1] and synthesis of Rubisco and other photosynthetic proteins [2]. However, bioavailability of P often also limits productivity of terrestrial and aquatic ecosystems [3], including in wet tropical forests [4]. Stocks, chemical forms and availability of P change with ecosystem development and succession processes determining ecosystem properties along spatial and temporal gradients [5,6,7,8]. Lowland tropical forest trees tend to experience long-term low P bioavailability, so it is likely that species have evolved responding and adapting to P-limitation and thus was reflected in tree community composition. Indeed, a recent study in a Panamanian tropical forest showed that P-limitation of plant growth is species-specific, and growth of species adapted to low P availability is rapid [9].

Tropical forests are characterized by high levels of biodiversity and aboveground biomass at a range of spatial scales; however, the spatial distribution of plant species and soils is highly variable within these forests, despite the tropical rainforest presented very often general characteristics, such as high productivity, rapid nutrient turnover, highly weathered soils and low soil pH [10]. Variations in climate, soil traits and gradients, and topography are drivers of diverse plant communities and soil processes that interact to produce high levels of biodiversity [11]. Factors, such as geological history [12], soil type [13,14] and trait [15,16], soil nutrient availability gradients [14,17,18,19], micro-site singularities [20], topography [21] and slope differences [22], disturbance and regeneration regimes [23] and species-specific responses to herbivory [24] are key drivers of species coexistence and tree diversity in the tropics.

Understanding the allocation of P to different biologic functions, for example, growth, energy transfer and storage, in sympatric tree species in a P-limited ecosystem will clarify strategies and mechanisms of species niche segregation and intra-species avoidance of competition. This can provide new essential clues that drives the high levels of tree diversity in tropical forests.

Shifting leaf allocation of P is a key response mechanism to low soil P availability [25]. Foliar P is functionally divided into four major fractions, comprising metabolic P—which includes low-molecular-weight phosphate esters (ADP, ATP and sugar phosphates)—and inorganic phosphate (P_i_); nucleic acid P, most of which is contained in ribosomal RNA; structural P in membrane phospholipids; and, residual P in phosphorylated proteins and unidentified residues [25,26,27]. Of these, metabolic P is important in the study of P-limitation, due to the key roles of P-containing metabolites in the Calvin–Benson cycle, where insufficient metabolic P may limit maximum photosynthetic rates [28]. Nucleic acid P, which generally represents 40%–60% of the organic P pool in leaves [29], is mostly (>85%) contained in RNA, particularly ribosomal RNA (rRNA) in which high P-allocations sustain rapid protein synthesis that is required for growth and photosynthesis [25]; thus, there tends to be a positive correlation between rRNA and protein content, as well as with growth rate, over a range of taxa [30]. Structural P accounts for 10%–20% of all foliar P [31] and is mainly contained in phospholipids that are an essential component of plasmalemma and organelle membranes. The fourth fraction, residual P, may represent 20% of total foliar P in tropical trees [25] and is probably mostly contained in phosphorylated proteins. Concentration of residual P tends to be relatively constant, because phosphorylated proteins are essential for many metabolic processes; however, under extreme P-limited conditions, phosphatases may trigger dephosphorylation of proteins [31] that leads to a reduction in residual P concentrations.

^31^P nuclear magnetic resonance (NMR)-based metabolic profiling allows the quantitative and qualitative analysis of organic-P from molecules involved in biologic functioning in plants and may demonstrate species differences in proportional P-use among plant functions. This methodology has been particularly used for molecular-level characterization of leaf organic P [32,33,34,35].

Here, we used ^31^P-NMR-based metabolic profiling to study species-specific P-allocation to different biologic functions in contrasting plant communities along soil–topographic gradients to understand drivers and mechanisms of niche differentiation related with species-specific P-use. Specifically, we tested the hypotheses that (1) distribution and proportions of P-molecular compounds are species-specific, and (2) there is a link between concentration and ratios of different P-metabolite groups and plant functional traits.

## 2. Materials and Methods

### 2.1. Study Area

French Guiana lies between 2°10′ and 5°45′ N and 51°40′ and 54°30′ W, where 97% of the region is covered by lowland wet tropical forest [36]. The dry season, which extends from September to November, is associated with the displacement of the inter-tropical convergence zone. Mean annual temperature is 25.8 ± 2 °C, with mean daily variations of 7 and 10 °C in the rainy and dry seasons, respectively [37,38]. Study sites were in two old-growth rainforests, one at the Paracou Research Station (5°18′ N, 52°53′ W) and the other at Nouragues Research Station (4°05′ N, 52°40′ W), where mean annual rainfall is 3160 and 2990 mm, respectively [37,38]. Three topographic positions were selected at each study, comprising hilltop (top), mid-slope at an intermediate elevation (slope) and lower slope at low elevation, just above the creek (bottom).

### 2.2. Study Plots

**Figure 1:**
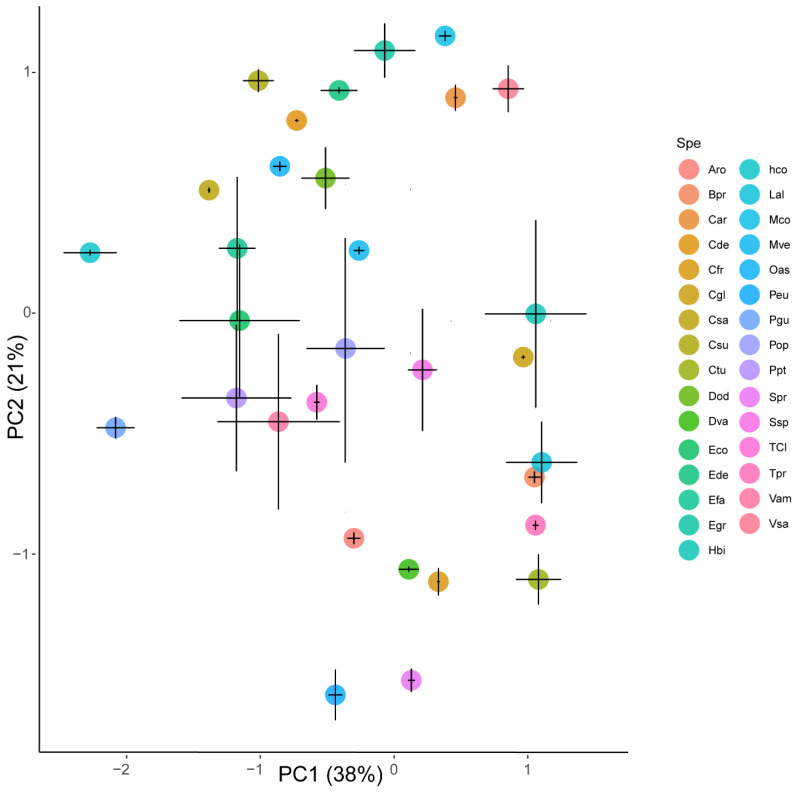

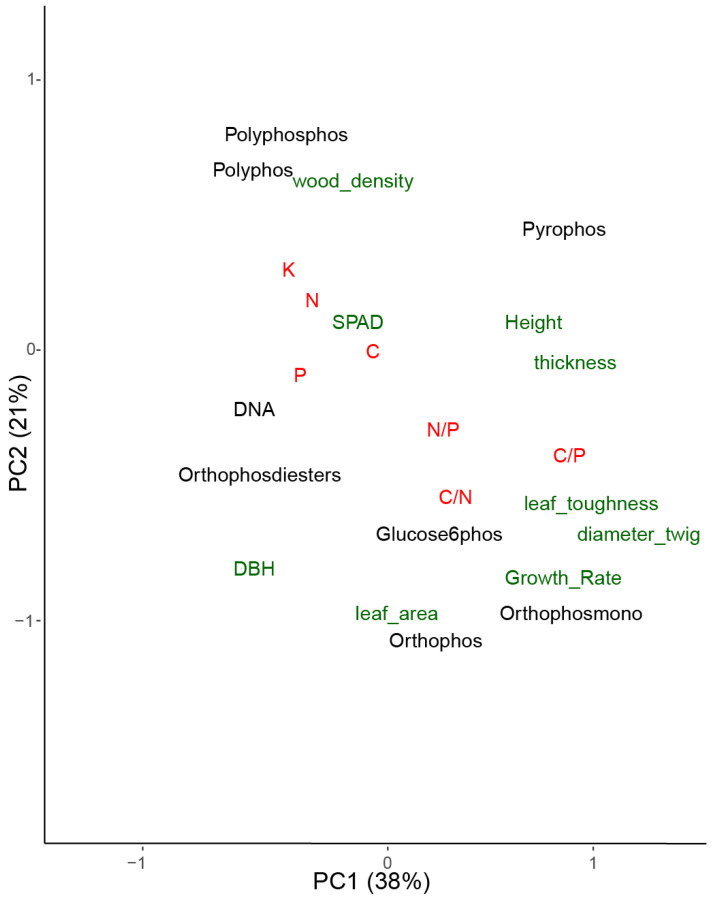
Principal components analysis (PCA) of P organic compounds and elements in leaf material among (**A**) species and (**B**) variables. In panel B, metabolomic families are in black text; ecophysiological variables are in green text; and stoichiometric variables are in red text. Polyphos—polyphosphate; pyrophos—pyrophosphate; polyphosphos—polyphosphonate; orthophosphate (orthophos), orthophosphate monosester (orthomono), orthophos—orthophosphate-diester; glucose6phos—glucose-6phosphates; SPAD—chlorophyll content; C—carbon; N—nitrogen; K—potassium; P—phosphorus. the closest tree species to each sampling point with the corresponding S.D. (B). The abbreviation of the legend refer to the closest tree species: Aro—*Aniba rosaeodora;* Bpr—*Bocoa prouacensis*; Car—*Chrysophyllum argenteum*—Cde—*Capiro decorticans*; Cfr—*Catostemma fragrans*; Cgl—*Caryocar glabrum*; Csa—*Chrysophyllum sanguinolentum*; Csu—*Carapa surimensis*; Ctu—*Chimarrhis turbita*; Dod—*Dipteryx odorata*; Dva—*Drypetes variabilis*; Eco—*Eschweilera coriacea*; Ede—*Eschweilera decolorans*; Efa—*Eperua falcata*; Egr—*Eperua grandiflora*; Hbi—*Hirtella bicornis*; hco—*Hymanea courbaril*; Lal—*Licania alba*; Mco—*Moronobea coccinea*; Mve—*Micropholis venulosa*; Oas—*Oxandra asbeckii*; Peu—*Pouteria eugeniifolia*; Pgu—*Paloue guianensis*; Pop—*Protium opacum*; Ppt—*Pradosia ptychandra*; Spr—*Sterculia pruriens*; Ssp—*Sterculia speciose*; TCl—*Tovomita clusiaceae*; Tpr—*Talisia praealta*; Vam *Vouacapoua america*; Vsa—*Vochysia sabatieri*.

We established four 20 × 20 m plots at each topographic position; distance between plots, which were in the vicinity of undisturbed long-term (30 years) monitoring plots, was 10–200 m (both in Paracou and Nouragues stations) (Figure 1). Sand content of soils was higher, and clay content was lower at the lower slope plots than in the hilltop and mid-slope plots [38].

### 2.3. Sample Collection

We collected leaves from 199 trees of 31 species in the wet season (June) of 2015. The trees were representative of the whole rainforest of the French Guiana and of the two studied sites, Paracou and Nouragues. We collected leaves always in the same position and always of similar age (mature middle-age leaves) with the help of professional climbers. Samples used in metabolomic analyses require rapid processing and appropriate storage [39,40,41,42], so we placed about 2 g of leaf tissue per sample immediately into a paper container that was then frozen in liquid nitrogen and transported to the laboratory.

Frozen leaves were lyophilized and stored in paper containers at −80 °C; then samples were ground to a fine powder using a ball mill at 1500 rpm for 3 minutes and stored at −80 °C prior to analysis extraction.

### 2.4. Nutrient Pools

Leaf subsamples were pulverized in a ball mill (MM400, Retsch, Haan, Germany) for the analysis of elemental composition. Between 0.15 and 0.2 g of leaf was weighed with a microbalance (MX5 Mettler Toledo, Columbus, OH, USA) for the determination of C and N contents by combustion coupled to an isotope ratio mass spectrometer at the Stable isotopes facility (UC Davis, Davis, CA, USA). P and K contents were determined by diluting 0.25 g of soil with an acid mixture of HNO_3_ (60%) and H_2_O_2_ (30% p/v) and digested in a microwave oven (MARS Xpress, CEM Corporation, Matthews, NC, USA). The digested solutions were then diluted to a final volume of 50 mL with ultrapure water and 1% HNO_3_. Blank solutions (5 mL of HNO_3_ with 2 mL of H_2_O_2_ with no sample biomass) were regularly analyzed. The content of each element was determined using inductively coupled plasma/optical emission spectrometry (ICP-OES Optima 4300DV, PerkinElmer, Wellesley, MA, USA). We used the standard certified biomass NIST 1573a to assess the accuracy of the biomass digestion and analytical procedures.

### 2.5. One Dimensional ^31^P-NMR Analysis

Standard 1D ^31^P-NMR was used to quantify concentrations of the main organic and inorganic P classes, comprising as DNA, total diesters and monoesters, phosphonates, pyrophosphate and polyphosphate. Phosphorus was extracted by shaking 1.5 g of dry and ground leaf from each composite leaf sample in 30 mL of a solution containing 250 mM NaOH and 50 mM Na2EDTA (ethylenediaminetetraacetate) for 4 h [43,44,45]. Then, extracts were centrifuged (30 min, 14,000× *g*), and 23 mL of resulting supernatant was frozen at −80 °C overnight and lyophilized. Lyophilization yielded 750 ± 50 mg of material, 80 mg of which was redissolved in 640 μL (1:8 *w/v* ratio) of a solution containing 530 μL of D_2_O, 10 μL of 14.2 M NaOD and 50 μL of 16 mM methylene diphosphonic acid (MDPA) trisodium salt (CH_3_O_6_P_2_Na_3_, Sigma-Aldrich, St. Louis, MO, USA, product number M1886). The MDPA was a reference for the quantification of individual P compounds, where each 50 μL spike contained 50 μg of P. The redissolved solution was vortexed for 2 min and subsequently centrifuged for 5 min at 10,000 rpm; then 560 μL of the solution was transferred to a 5-mm NMR tube for analysis. During the extraction, we continuously maintained the pH at 8. We used all the samples at the same pH to compare all the samples in the same condition. Subsequent organic phosphorus extraction in NaOH–EDTA improved spectral resolution in solution ^31^P-NMR spectroscopy. Spectra were obtained using a Bruker Avance III 600 MHz spectrometer (Bruker, Germany) operating at 161.76 MHz for ^31^P; NaOH–EDTA extracts were analyzed using zgpg30 pulse sequence, with a relaxation delay of 2.0 s, an acquisition time of 0.9 s, broadband proton decoupling, 8k scans were acquired per sample and using 64 K time domain data points, lasting each run an overall time of 4 h 40 min. Spectra were processed with a line broadening of 2 Hz, and chemical shifts were determined in parts per million (ppm) relative to an external standard of 85% orthophosphoric acid (H_3_PO_4_). Identification of the 5target P classes was based on chemical shifts and previous reported data [32,36]. Spectral processing was done using TopSpin 2.0 software. After a peak picking process, peak areas were calculated by deconvolution and integration of individual peaks. Concentration of P-containing compounds (mg P kg^−1^ air dried leaf) were calculated using the known concentration of spiked MDPA.

^31^P-NMR is an NMR technique that does not a require isotope labeling and highly sensitive with NMR detection. Intensity in ^31^P spectra signals was assigned to P types by integrating across the broad chemical shift regions of −21.5 to −18.5 ppm for non-terminal polyphosphate (poly-P), −5.3 to −4.8 ppm for pyrophosphate (pyro P), −4.8 to −4.0 for terminal poly P, which is mainly assigned to inorganic pyrophosphate and polyphosphates, −1.5 to 2.5 for diester-P and 2.5 to 7 ppm for orthophosphate (ortho-P and monoester-P). Deconvolution, which was then used to determine the intensity of up to 16 resonances in the ortho-P and monoester-P region, was initiated by manually identifying the chemical shift of peaks and shoulders. Chemical shifts of corresponding resonances varied only slightly between samples, and the largest variation was for orthophosphate resonance (range 5.56–5.74 ppm). This large variation most likely reflects sensitivity of orthophosphate chemical shifts to slight differences in pH between samples [46].

### 2.6. Statistical Analyses

Differences in leaf P-compounds between species tested using PERMANOVA [47] of the NMR data for each tree from which a leaf was collected, using Euclidean distance of species as a fixed factor. The number of permutations was set at 2000. We scaled normalized areas of metabolite peaks and then characterized and visualized differences among species and P compounds using principal components analysis (PCA), partial least squares discriminant analysis (PLSDA) and Pearson’s correlation were used to test the degree of correspondence between the 1D NMR measurements and the relationship between elements and P organic compounds.

Statistical procedures were performed using R v2.12 (www.r-project.org) Core software using the SEQKNN, DOBY, PHEATMAP, VEGAN, FACTOEXTRA, FACTOMINER, DPLYR and MIXOMICS packages. Presented data are means ± SEM (Standard error of the mean), unless otherwise stated.

## 3. Results

### 3.1. Phosphorus Compounds Identification

On average, ^31^P-NMR spectra of NaOH–EDTA extracts (and relative proportions,%) from the leaf samples were characterized thus: monoester at 3.4 to 5.4 ppm (c. 25%), unhydrolyzed diesters at −1 to 2.3 ppm (c. 20%), DNA at −0.3 ppm (c. 20%), polyphosphate at −5.6 to −3.8 (c. 10%), inorganic orthophosphate (hereafter called ‘phosphate’ at 5.7 to 6.5 (c. 10%), pyrophosphate (pyrophos) inorganic form of P at 0.5 to 0.6. ppm (c. 5%) and glucose-6phosphates (glucose6phos) at 5.3 to 5.4 ppm (c. 5%). However, there were three main resonance areas in the spectra of acid-insoluble compounds: P in monoesters, phospholipids (orthophosphate diester) and DNA. Signals from nucleic acids (DNA −0.37 ppm) and phospholipids were differentiated in the orthophosphate diester (orthophosdiester) region and identified in a leaf sample. Inorganic (polyphos) and organic polyphosphates were differentiated by the presence of a signal at −9 ppm from the α phosphate of organic polyphosphates (polyphosphos). Some orthophosphate monoesters, which were mainly represented by RNA-derived mononucleotides and phosphatidyl choline, degraded rapidly to orthophosphate diesters in NaOH–EDTA; DNA and other phospholipids were more stable [32,36].

### 3.2. Phosphorus Compounds and Elements Among Species

There were species differences in P organic compounds profiles (pseudo-*F* = 3.08; *R*^2^ = 0.67, *p* < 0.001), where 67% of the variability was explained by species (Table 1).

Concentrations of P in the tree leaf material varied from 0.73 to 2.10 mg kg^−1^ (Table 2), where NaOH–EDTA extracted P represented 65%–90% of total P; 15%–40% was inorganic P that was almost completely lost during NaOH–EDTA extraction and loss of P_org_ did not exceed 15%–20%. Proportions of monoester and diester-P varied among tree species (Table 2). The highest proportion of diesters (55%) was recorded in *Calostemma fragrans*, *Sterculia pruriens*, *S. speciosa*, *Protium opacum*, *Aniba rosaeodora* and *Drypetes variabilis*, while the lowest (10%) was found in *Chrysophyllum argenteum*, *Tovomita clusiacege*, *Eschweilera decolorans*, *Capirona decorticans*, *Paloue guianensis*, *Dipteryx odorata* and *Talisia praealta*. The proportion of phospholipid-P exceeded 30% in extracts from *Tovomita clusiacege* and was 10% in extracts from the other species (Table 2).

### 3.3. Phosphorus Compounds and Elements

PCs 1 and 2 of the PCA of P organic compounds and elements concentration in leaf material among species represented 59% of the variation and showed several separations among species (Figure 1A). The results of the PLS–DA were very similar to the results of PCA (Figure 1). Nutrients were a key driver, as indicated by higher N and P concentrations and lower C:P and C:N ratios along PC1 axis, whereas variation in PC2 was associated with distinct identified P family components, with observed gradient along this axis from P families associated with energy transference and genetic information and protein synthesis to organic P-family (polyphosphates) associated with energy reserves (Figure 1B).

## 4. Discussion

Our results support our hypothesis that P metabolite profiling differ among species, reflecting niche differentiation in the P-use. We observed that species tended to be characterized by the composition in leaf tissue of main functional groups of metabolic P-containing molecules and elements, indicating species use of different forms of P drive is species-specific diversity in the studied tropical wet forest studied sites. We observed clear species niche differentiation along PC2 (Figure 1B) along the gradient constituted from high-anabolic-energy P-use and low P-storage compounds to low-anabolic-energy P-use and high P-storage compounds; however, there were no clear patterns of this relationship with total P concentration or between total foliar P concentration and the foliar P metabolome. One possible explanation for these contrasting results may be the lack of direct comparison, because the P-metabolome was analyzed using water extracts, whereas P concentrations were determined from leaf structural tissues, where there are species variations in light use or anti-herbivore strategy, may result in species with higher levels of P use for active metabolism, but not necessarily higher levels of total P concentration. Leaves directly exposed to sunlight tend to be sclerophyllous and contain low P concentrations; however, most P is allocated to energy and anabolic metabolism due to the high photosynthetic activity and levels of energy storage.

Our results show that proportional and functional use of P varies with species, indicating niche partitioning. Similarly, previous studies in this tropical forest have observed that general foliar metabolomic profiles show clear species-specific signatures that indicate functional niche separation in dominant tree species [48]. Our results indicate that microsite differences in resource bioavailability create variations in niche conditions that drive multiple species coexistence at small spatial scales.

Classical species niche hypotheses are based on the concept that potential competing sympatric species have varying optimum basic abiotic resource requirements (light, water and nutrients) and contrasting biotic relationships [49]. Previous studies have identified associations between species assemblages and habitat conditions in tropical rainforests [50,51,52,53], such as variations in leaf properties (parental material, drainage), leaf nutrient availability [14] and topography (slope steepness and orientation, margins with water courses), that are consistent with niche theory. Extensions of the classical ecological niche hypothesis, such as the biogeochemical niche hypothesis, claim that species tend to reach an optimal chemical composition that is linked to a singular optimal function (homeostasis) and allows niche occupation [54,55,56,57,58]. Thus, this study provides evidence for the use of a fundamental resource, such as the P, in the characterization of species niche space. This approach may be powerful in sites with limited P, such as in rainforests, where a P-metabolic niche would reflect species-specific functional adaptions along natural gradients that drive bioavailability, total concentration and content of P as consequence of long-term different sympatric species co-evolution that should have optimized community use and conservation of the scarce amounts of P. Tropical trees may occupy finely subdivided niches [59], but there is little direct evidence for a measurable variable that reflects overall functional differences among sympatric species. Our results clearly indicate that species niche specificity may be determined, at least partially, by analyzing variance in the P-metabolic profile among sympatric species, together with other potential sources of environmental variability, such as micro-site conditions and topography. It is likely, therefore, that environmental factors, such as topography and leaf physicochemical properties, could be related to species-specific use of P. That is also consistent with the significant change of P-use and niche differentiation across distinct space situation due slope at microscale spatial level and also to medium spatial distances in this pristine rainforest area in French Guiana. Implies a continuum of strategies form an extreme of high growth rates, high leaf area, low wood density and high P-use in anabolic and energy store and transference to another extreme strategy with low growth rate and leaf area, high wood density and high P-reserve compounds.

## 5. Conclusions

This study found large differences in P-metabolite profiling among sympatric tree species in French Guianese rainforests. ^31^P-NMR-based metabolic profiling analysis showed tree species that contained high concentrations of P-organic compounds related to energy, information and protein synthesis had low concentrations of P-organic compounds related to P-storage; in other hand the reverse, were P-organic compounds related to P-storage had higher concentration had low concentration of P-organic compounds related to energy, information and protein synthesis was true.

These results are consistent with the niche theory and demonstrate the usefulness of P-metabolome as an analytical tool to determine niche differences in the functions of plant species in a community under limited nutrient bioavailability. It is likely that metabolomic niches in old, undisturbed tropical forest ecosystems are associated with the optimal use-exploitation of environmental niches (light conditions, water and nutrient availability, and/or specific biotic relationships) acquired through evolutionary processes. In tropical forests on P-poor soils, species exhibit morphologic, physiological, molecular and biochemical adaptions, where P-use forms are related to basic functional traits, such as growth rate, leaf area and wood density.

Described results strongly suggested that existed a continuum of strategies form from the species to relate an extreme of high growth rates, high leaf area, low wood density and high P-use in anabolic and energy store. Another extreme strategy with low growth rate and leaf area, high wood density and high P-reserve compounds.

The results were also consistent with the significant change of P-use and niche differentiation across distinct space situation due to slope at microscale spatial level and to medium spatial distances in this pristine rainforest area in French Guiana.

## Figures and Tables

**Table 1:** Test for species differences in phosphorus (P) organic compound content in leaf material.

Source	*df*	SS	MS	*F*	*R* ^2^	*p*
Species	33	10206396	309,285	3.08	0.67	0.001
Residuals	49	4914344	100,293	0.33		
Total	82	15120740	1			

**Table 2:** Profile of P-compounds in leaf material of tree species. ^31^P NMR spectra of NaOH–EDTA extracts (and relative proportions, %) from the leaf samples were characterized.

Species	Total P (%, D.W.)	Total C (%, D.W.)	Total N (%, D.W.)	C:N	C:P	N:P	K (%, D.W.)	Phosphorus Species Distribution, % of Total P in Extract							
								Polyphos	Orthophos	Glucose-6phos	Orthophos-mono	Orthophos-diesters	DNA	Pyrophos	Poly-P
*Licania alba*	0.04	48.59	1.44	34.7	1151.91	33.17	0.35	3.55	7.11	0	14.12	28.57	16.57	17.30	12.79
*Vouacapoua americana*	0.07	50.2	1.99	25.6	687.46	26.84	0.67	1.67	8.57	24.17	22.41	15.52	6.75	13.92	6.99
*Chrysophyllum argenteum*	0.03	49.7	1.34	37.2	1435.60	38.59	0.40	40.25	0	0	11.77	11.57	16.82	0	19.60
*Oxandra asbeckii*	0.05	48.1	1.94	24.8	1042.83	42.13	1.69	24.25	63.24	0	0.65	1.30	10.04	0	0.52
*Hirtella bicornis*	0.04	47.1	1.32	35.9	1215.23	33.94	0.75	0	0	0	26.38	13.88	9.03	25.13	25.58
*Tovomita sp.*	0.07	49.3	1.61	30.6	670.28	21.91	0.72	0	6.65	16.55	6.99	16.12	22.00	0	31.70
*Moronobea coccinea*	0.03	48.8	1.28	38.4	1469.22	38.49	0.27	5.88	0	0	1.82	46.14	23.60	0	22.55
*Eschweilera coriacea*	0.07	50.0	1.71	30.7	975.27	30.72	0.50	1.60	16.35	10.46	17.44	22.40	16.29	8.91	6.56
*Hymanea courbaril*	0.12	48.5	1.91	25.3	412.80	16.29	0.87	42.19	38.05	3.09	0	0	11.35	5.31	0
*Eschweilera decolorans*	0.05	50.0	1.77	28.2	1103.39	39.14	0.58	32.27	0	0	9.36	27.51	6.00	0	24.85
*Capirona decorticans*	0.05	48.7	2.08	23.4	1077.96	46.08	0.41	0	0	0	11.37	53.25	32.62	0	2.77
*Pouteria eugeniifolia*	0.08	48.6	2.89	16.9	650.54	38.58	0.69	0	35.00	0	15.72	19.61	19.61	0	10.06
*Eperua falcata*	0.08	49.6	1.70	30.1	687.85	23.49	0.63	7.37	0	6.41	25.85	43.73	10.22	0	6.41
*Catostemma fragrans*	0.03	25.2	0.90	14.0	455.01	16.29	0.28	0	49.45	2.50	28.06	0	20	0	0
*Caryocar glabrum*	0.05	52.4	1.34	39.2	1009.50	25.75	0.36	0	22.91	0	20.06	20.06	32.87	3.59	0.51
*Eperua grandiflora*	0.04	50.5	1.39	37.8	1289.63	34.47	0.34	6.39	7.22	6.46	25.13	19.73	12.13	18.70	4.25
*Paloue guianensis*	0.05	52.0	1.76	29.5	1086.24	36.77	0.22	2.21	0	19.81	7.14	21.05	21.63	28.16	0
*Dipteryx odorata*	0.06	49.4	1.38	35.9	891.48	24.81	0.89	0.31	12.79	8.35	4.57	16.65	3.13	0	54.19
*Protium opacum*	0.06	47.7	1.25	38.5	856.32	22.39	0.67	14.49	32.93	0	40.44	0.80	7.12	0	4.21
*Talisia praealta*	0.04	48.9	1.23	39.9	1203.11	30.12	0.21	0	0	0	10.14	18.78	18.78	23.31	28.98
*Bocoa prouacensis*	0.04	48.8	1.66	31.0	1471.96	47.02	0.35	0	27.66	14.21	14.21	4.61	2.66	0.46	36.19
*Sterculia pruriens*	0.07	46.9	1.42	33.2	686.14	20.66	1.14	0	19.99	22.74	44.54	0	12.73	0	0
*Pradosia ptychandra*	0.08	46.7	1.79	27.1	727.94	25.16	1.57	10.66	0	0	28.35	17.79	10.19	0	33.01
*Aniba rosaeodora*	0.05	48.2	1.44	33.5	1019.02	30.46	0.97	0	0	0	50	30	20	0	0
*Vochysia sabatieri*	0.04	47.31	1.22	39.03	1357.56	34.89	0.23	0	2.20	0	30.08	41.23	14.48	2.68	9.33
*Chrysophyllum sanguinolentum*	0.06	48.02	2.42	19.85	816.72	41.14	1.47	0	0	0	18.18	59.02	13.77	0	9.03
*Sterculia speciosa*	0.05	48.63	1.35	36.10	1020.57	28.27	0.86	1.07	12.07	7.77	59.09	10	10	0	0
*Carapa surinamensis*	0.06	48.49	1.41	34.61	816.65	23.59	0.50	30.42	0	13.52	0	21.87	22.02	0	12.17
*Chimarrhis turbinata*	0.05	47.42	1.99	23.87	941.32	39.24	0.23	0	15.06	22.14	15.74	17.90	4.48	24.69	0
*Drypetes variabilis*	0.05	45.13	1.45	31.15	847.58	27.14	1.03	22.84	15.94	3.22	37.99	16.00	4.00	0	0
*Micropholis venulosa*	0.04	47.48	1.90	25.04	1149.58	45.91	0.65	0	0	0	27.64	23.10	28.69	0	20.57
